# Trend of disease burden and risk factors of breast cancer in developing countries and territories, from 1990 to 2019: Results from the Global Burden of Disease Study 2019

**DOI:** 10.3389/fpubh.2022.1078191

**Published:** 2023-01-16

**Authors:** Linlin Lv, Binggong Zhao, Jie Kang, Shujing Li, Huijian Wu

**Affiliations:** ^1^Key Laboratory of Protein Modification and Disease, School of Bioengineering, Dalian University of Technology, Dalian, Liaoning, China; ^2^Department of Pharmacy, First Affiliated Hospital of Dalian Medical University, Dalian, Liaoning, China

**Keywords:** breast cancer, Global Burden of Disease, developing country, risk factors, mortality, incidence

## Abstract

**Background:**

The incidence, mortality, burden of disability-adjusted life years (DALYs), and attributable risk factors of breast cancer vary significantly by country or region, particularly between developing and developed countries. This study aimed to analyze breast cancer development trends in developing countries based on the influence of the different sociodemographic indices (SDIs) and World Bank (WB) income-level disease data from 1990 to 2019.

**Methods:**

Data on the annual incidence, mortality, DALY, years of life lost (YLL) prematurely, years lived with disability (YLD), and age-standardized rate (ASR) of breast cancer from 1990 to 2019 in different countries and territories were obtained from the 2019 Global Burden of Disease (GBD) Study. A comparative risk assessment (CRA) framework was used to analyze the general risk factors.

**Results:**

The global age-standardized incidence rate (ASIR) gradually increased from 21.44 per 100,000 population in 1990 to 24.17 per 100,000 population in 2019. It rose precipitously to 2.91- and 2.49-fold, respectively, for countries with middle SDIs and low-middle SDIs. The ASIR of breast cancer was increasing in the lower-middle-income levels in WB, with an estimated annual percentage change (EAPC) of 0.29 [95% uncertainty interval (UI): 0.20–0.37] and reduced income (EAPC of 0.59 [95% UI: 0.53–0.65]). The Solomon Islands and the United Arab Emirates observed the most significant increase in the magnitude of deaths from breast cancer cases. Compared to the death cases of 1990, percentage changes increased separately by 1,169 and 851%. Compared to developed areas, breast cancer-related deaths increased rapidly in developing regions, especially among the middle-aged and elderly groups. Meanwhile, the long-term burden of breast cancer was ever expanding. Of all the GBD regions, Oceania had the youngest age distribution. The deaths in the young and middle-aged groups accounted for 69% in 1990 and 72% in 2019. Percentage changes in deaths from the seven risk factors in low- to middle-SDI regions increased significantly over time across all age groups. However, a diet with high red meat and high body mass index (BMI) accounted for the most considerable increase in the magnitude.

**Conclusion:**

Public health policy regarding breast cancer is fundamental in low- and medium-income countries. The development and adoption of cost-effective screening and therapeutic solutions, the mitigation of risk factors, and the establishment of a cancer infrastructure are essential.

## 1. Introduction

Cancer is the second leading cause of death worldwide, placing a significant economic burden on public health systems (1). However, incidence and mortality rates, as well as the burden of disability-adjusted life years (DALYs), of cancer vary significantly by country or region, particularly between developing and developed countries (2). In addition, various factors, including ecological, environmental, demographic, and cultural factors, contribute to a heterogeneous cancer incidence, mortality, and DALY burden (3).

Breast cancer is the leading cause of death among women, and its incidence ranks first among the most prevalent tumors in women. According to the World Health Organization (WHO), ~2.26 million new cases of breast cancer in women were diagnosed worldwide in 2020, accounting for one in every eight cancer patients—making breast cancer the world's top most prevalent type of cancer (4).

Over the past three decades, breast cancer incidence and mortality have increased in developing and developed countries (5). The global incidence of breast cancer among women is predicted to increase by 2035 (6). Despite improvements in breast cancer survival, they differ significantly in different countries (7). This is due to factors such as ineffective screening, detection, and cost-effective treatment in the early stages of the disease (8). Some studies showed that the global burden of breast cancer among women continues to increase, particularly in developing countries. Along with economic growth and changes in the wave of industrialization that brought about increased labor force participation by, education of, and emancipation of women, there were changes in reproductive patterns such as younger menarche ages, shorter breastfeeding periods, later marriage, and lower levels of equality. In addition to these changes, changes in diets, lack of physical activity, and leading a sedentary lifestyle are characteristics of westernized lifestyles, increasing the risk of breast cancer in women who have been less burdened in developing countries up to date (9). Therefore, developing countries and regions need to invest in breast cancer prevention, promote public awareness of breast cancer risk factors, and develop policies to prevent this disease (6).

## 2. Objectives

The study aimed to analyze breast cancer development trends in developing countries based on the influence of different sociodemographic indices (SDIs) and World Bank (WB) income levels on breast cancer incidence, mortality, and DALYs from the 2019 Global Burden of Disease (GBD) data from 1990 to 2019. Based on GBD in breast cancer, it could help provide timely assessment and guidance to address these serious disease problems.

## 3. Methods

### 3.1. Data source

Study data were obtained using the Global Health Data Exchange (GHDx) query tool (http://ghdx.healthdata.org/gbd-results-tool), an online data source tool. All available epidemiological data were adopted in this ongoing global collaboration to assess health losses caused by 369 diseases and 87 risk factors across 204 countries and 21 GBD regions (1). Based on the social development index, countries are classified into five SDI quintiles (high, high-middle, middle, low-middle, and low). Regarding income levels, the WB is divided into four levels, namely high income-level, upper-middle income-level, lower-middle income-level, and low-income-level regions. Furthermore, the world is divided into 21 GBD regions by geographic locations.

Based on the GBD Study 2019, we gathered the annual incidence, mortality, DALY, years of life lost (YLL) prematurely and years lived with disability (YLD), and age-standardized rate (ASR) of breast cancer from 1990 to 2019. DALYs can be used to estimate the global cancer burden, YLLs can be used to estimate the early mortality burden related to breast cancer, and YLD reflects the number of years living with short- or long-term health losses, weighted by disability severity. In addition, DALY can also be calculated by combining YLL and YLD measures of disease burden (10).

### 3.2. Estimation methods

In the GBD Study 2019, a comparative risk assessment (CRA) framework was used to calculate the burden attributable to risk factors, which is based on the amount of disease burden that can be reduced by reducing exposure to specific risk factors to the theoretical minimum risk exposure level (TMREL) (10). We estimated the mean exposure level using the Bayesian meta-regression model (DisMod-MR 2.1) and the spatiotemporal Gaussian process regression model (ST-GPR) based on exposure levels revealed in a population-based survey or report.

A detailed description of a framework for assessing GBD risk factors is presented in the 2019 GBD risk factor study (10). We determined the relative risk and outcome value using high-quality literature studies after confirming their correlation. Finally, in this study, alcohol consumption, high fasting plasma glucose (FPG), high body mass index (BMI), a diet high in red meat, low physical activity, secondhand smoke, and smoking were identified as the seven major risk factors.

### 3.3. Attributable burden and statistical analysis

To quantify breast cancer, incidence and mortality, ASRs, DALYs, and the estimated annual percentage changes (EAPCs) were calculated. The calculation formula of ASRs (per 100,000 population) was developed based on a previous study (11). The formula for calculating EAPC is as follows:


$$ASR=\frac{\sum_{i=0}^{A}aiwi}{\sum_{i=1}^{A}wi}\times 100,000$$

(ai, denotes the ith age class, and the number of persons (or weight) (wi) in the same age subgroup i of the selected reference standard population).

Age-standardized rate trends can be an important indicator of changing disease and risk factor patterns. To reflect such trends in time, we used a widely used ASR-EAPC measurement (12, 13). EAPC is a concept developed to describe ASR trends within a specified time interval, assuming a linear relationship between ASR and natural logarithm. Thus, *Y* = α + β*X* + ε, *Y* represents ln (ASR), *X* represents the calendar year, and ε represents the error term. According to this formula, β determines whether ASR trends are positive or negative. The formula for calculating EAPC is as follows:


$$\begin{array}{l} EAPC=100\times \left[exp\left(\beta \right)-1\right]. \end{array}$$

The linear model provides 95% confidence intervals (CIs) (14, 15). ASR tends to rise when EAPC and the lower CI are positive. Conversely, in a downward trend, ASR exhibits a negative EAPC and an upper CI.

We used R (version 4.0.4, R core team) for all statistical analyses. *P*-values of < 0.05 were considered statistically significant.

## 4. Results

### 4.1. Breast cancer-related incidence cases and mortality in developing regions

Globally, the number of breast cancer cases increased to 2.00 million (95% uncertainty interval (UI): 1.83–2.17) in 2019 (Table 1). Therein, there were 1.98 million (95% UI: 1.81–2.15) women with breast cancer. Meanwhile, the global age-standardized incidence rate (ASIR) gradually increased over the past 30 years, from 21.44 per 100,000 population (95% UI: 20.65–22.10) in 1990 to 24.17 per 100,000 population (95% UI: 22.11–26.24) in 2019, with an EAPC of 0.33 (95% UI: 0.28–0.38). Women and men showed similar trends. In the past three decades, all SDI quintiles have experienced an increase in breast cancer incidence. Middle and low-middle SDI quintiles countries experienced a 2.91- and 2.49-fold increase in breast cancer incidence, respectively. The middle SDI quintile showed the fastest growth in ASIR for breast cancer, with an EAPC of 1.95 [95% UI: 1.98–1.92], but decreased in the high SDI quintile over the past three decades, with an EAPC of −0.27 [95% UI: −0.37 to 0.18].

**Table 1:** The incidence and death cases and their age-standardized rates of breast cancer in 1990 and 2019, and its temporal trends from 1990 to 2019.

**Characteristics**	**1990**				**2019**				**1990–2019**	
	**Incidence** ** No. × 10^3^** ** (95% UI)**	**ASIR** ** per 100,000** ** (95% UI)**	**Deaths** ** No. × 10^3^** ** (95% UI)**	**ASDR** ** per 100,000** ** (95% UI)**	**Incidencev No. × 10^3^** ** (95% UI)**	**ASIR** ** per 100,000** ** (95% UI)**	**Deaths** ** No. × 10^3^** ** (95% UI)**	**ASDR** ** per 100,000** ** (95% UI)**	**EAPC** ** of ASIR** ** (95% UI)**	**EAPC** ** of ASDR** ** (95% UI)**
Global	876.99 (849.69–903.82)	21.44 (20.65–22.10)	380.91 (364.81–396.71)	9.80 (9.30–10.21)	2,002.35 (1,832.15–2,172.54)	24.17 (22.11–26.24)	700.66 (647.38–751.56)	8.62 (7.95–9.25)	0.33 (0.28 to 0.38)	−0.56 (−0.60 to −0.51)
**Sex**										
Male	9.37 (8.81–9.97)	0.539 (0.50–0.57)	5.89 (5.43–6.37)	0.37 (0.34–0.40)	25.14 (22.23–27.79)	0.65 (0.58–0.72)	12.10 (10.69–13.32)	0.33 (0.29–0.36)	0.91 (0.76 to 1.06)	−0.23 (−0.36 to −0.10)
Female	867.62 (840.40–894.76)	40.12 (38.78–41.33)	375.02 (358.98–390.82)	17.76 (16.93–18.51)	1,977.21 (1,807.61–2,145.21)	45.86 (41.91–49.76)	688.56 (635.32–739.57)	15.88 (17.07–14.66)	0.36 (0.31 to 0.42)	−0.51 (−0.56 to −0.46)
**SDI**										
High	431.08 (416.85–440.22)	43.20 (41.83–44.11)	137.44 (130.74–140.80)	13.55 (12.90–13.88)	678.94 (606.88–753.70)	41.22 (36.88–45.65)	167.55 (151.81–176.81)	9.05 (8.36–9.47)	−0.27 (−0.37 to −0.18)	−1.52 (−1.56 to −1.47)
High middle	228.48 (220.96–236.31)	20.93 (20.23–21.63)	104.54 (100.73–108.32)	9.98 (9.58–10.35)	516.51 (464.37–574.12)	26.00 (23.34–28.88)	165.93 (152.74–179.59)	8.31 (7.63–9.00)	0.68 (0.77 to 0.59)	−0.83 (−0.95 to −0.71)
Middle	125.97 (116.28–136.06)	10.66 (9.87–11.48)	72,639.6 (67,619.4–78,672.4)	6.67 (6.20–7.17)	493.15 (437.20–552.90)	18.52 (16.43–20.76)	184.79 (166.33–205.34)	7.30 (6.60–8.12)	1.95 (1.92 to 1.98)	0.29 (0.26 to 0.32)
Low middle	66.10 (58.24–74.33)	9.58 (8.39–10.74)	46.52 (40.56–52.30)	7.23 (6.24–8.14)	230.77 (202.83–259.30)	15.38 (13.54038–17.27)	127.41 (110.48–144.92)	8.94 (10.16–7.77)	1.54 (1.44 to 1.64)	0.63 (0.54 to 0.72)
Low	24.85 (20.72–28.76)	9.24 (7.66–10.79)	19.51 (16.31–22.90)	7.79 (6.47–9.30)	81.72 (71.26–93.26)	13.51 (11.93–15.22)	54.46 (47.49–620.57)	9.83 (8.59–11.12)	1.28 (1.22 to 1.34)	0.75 (0.71 to 0.80)
World bank income level	876.46 (849.17–903.28)	21.44 (20.65–22.10)	380.65 (364.57–396.46)	9.80 (9.30–10.21)	2,001.08 (1,831.00–2,171.13)	24.16 (22.1069–26.24)	700.15 (646.95–750.98)	8.61 (7.95–9.25)	−0.56 (−0.60 to −0.51)	0.32 (0.28 to 0.37)
World bank high income	515.08 (498.02–525.51)	42.24 (40.89–43.09)	169.90 (161.99–173.85)	13.68 (13.05–14.01)	807.38 (719.03–892.04)	41.13 (36.84–45.46)	208.02 (188.86–219.55)	9.35 (8.66–9.80)	−1.45 (−1.50 to −1.40)	−0.21 (−0.32 to −0.11)
World bank upper middle income	205.71 (190.77–221.83)	12.46 (11.61–13.40)	104.23 (96.58–112.32)	6.78 (6.30–7.28)	706.36 (618.57–807.16)	20.57 (18.03–23.50)	219.87 (197.74–244.69)	6.59 (5.91–7.33)	−0.18 (−0.28 to −0.08)	1.78 (1.71 to 1.84)
World bank lower middle income	137.99 (126.21–150.04)	11.56 (10.52–12.60)	93.10 (84.51–102.40)	8.38 (7.53–9.29)	437.28 (388.68–486.73)	16.45 (14.64–18.28)	238.60 (210.29–267.15)	9.56 (8.40–10.68)	0.29 (0.20 to 0.37)	1.09 (1.00 to 1.17)
World bank low income	17.68 (15.13–20.67)	10.58 (9.03–12.22)	13.42 (11.57–15.52)	8.63 (7.41–9.95)	50.06 (41.60–60.45)	13.96 (11.75–16.66)	33.66 (28.45–39.95)	10.29 (8.77–12.07)	0.59 (0.53 to 0.65)	0.94 (0.86 to 1.02)

ASIR, age-standardized incidence rate; ASDR, age-standardized death rate; CI, confidence interval; EAPC, estimated annual percentage change; UI, uncertainty interval.

From the WB income level, the incidence followed a global trend. The incidence of cases increased faster at the WB lower-middle- and upper-middle-income levels (2.43- and 2.17-fold, respectively) between 1990 and 2019. ASIR for breast cancer increased at the WB lower-middle-income level (EAPC of 0.29 [95% UI: 0.20–0.37] and at the low-income level (EAPC of 0.59 [95% UI: 0.53–0.65]. On the other hand, there was a decrease at the WB high-income level, with an EAPC of −1.45 [95% UI: −1.50 to −1.40] (Table 1).

Changes in disease burden are primarily impacted by changes in disease-associated mortality (16). Further analyses were conducted on the global and regional dynamics of breast cancer-related death burden during this period. Globally, the age-standardized death rate (ASDR) declined gradually from 1990 to 2019 (9.80 per 100,000 population [95% UI: 9.30–10.21] in 1990 and 8.62 [95% UI: 7.95–9.25] in 2019), with an EAPC of −0.56 [95% UI: −0.6 to −0.51]. Both men and women experienced similar trends. As SDI improved, total breast cancer-related deaths increased in the 1990s. However, during this period, breast cancer-related deaths have increased dramatically in the low-SDI region (Table 1).

Death rates and DALYs are used as indicators of the overall health burden of breast cancer. DALY consists of YLL and YLD. Over the past 30 years, there has been a significant increase in the incidence, mortality, DALYs, YLLs, and YLDs of all breast cancer-related cases. For instance, the total number of death cases increased steadily from 0.38 million (95% UI: 0.36–0.40) in 1990 to 0.70 million (95% UI: 0.65–0.75) in 2019 (Table 1). DALYs increased from 11.68 million (95% UI: 11.17–12.26) in 1990 to 20.63 million (95% UI: 19.04–22.17) in 2019 (Table 2). YLL and YLD in breast cancer were similar to DALYs, and the trend was upward. The global ASR for mortality, DALYs, and YLL gradually decreased, but YLD increased (Table 3).

**Table 2:** The case numbers of disability-adjusted life years and age-standardized rates disability-adjusted life years of breast cancer in 1990 and 2019, and its temporal trends from 1990 to 2019.

**Characteristics**	**1990**		**2019**		**1990–2019**
	**DALYs** **No**. × **10**^3^ **(95% UI)**	**ASDALR** **per 100,000(95% UI)**	**DALYs** **No**. × **10**^3^ **(95% UI)**	**ASDALR** **per 100,000(95% UI)**	**EAPC** **of ASDALR** **(95% UI)**
Global	11,681.06 (11,169.25–12,261.81)	275.32 (263.24– 288.77)	20,625.31 (19,043.05–22,174.40)	247.63 (228.68–266.08)	−0.50 (−0.56 to −0.45)
**Sex**					
Male	154.37 (142.70–166.95)	8.18 (7.54–8.82)	315.13 (278.55–349.29)	7.96 (7.03–8.80)	0.14 (−0.01 to 0.29)
Female	11,526.68 (11,021.13 −12,107.83)	524.87 (501.78–551.15)	20,310.19 (18,744.80–21,866.65)	473.83 (437.30–510.51)	−0.51 (−0.57 to −0.45)
**SDI**					
High	3,713.79 (3,586.31–3,845.10)	377.54 (364.84–390.67)	4,083.85 (3,816.26–4,354.65)	252.69 (237.71–269.32)	−1.53 (−1.58 to −1.48)
High middle	3,169.42 (3,050.87–3,304.08)	286.35 (275.39–298.36	4,570.25 (4,209.33–4,954.00)	230.43 (212.17–249.87)	−0.99 (−1.10 to −0.87)
Middle	2,506.04 (2,334.45–2,721.72)	202.41 (188.65–218.93)	5,923.36 (5,318.52–6,558.62)	219.85 (197.35–243.15)	0.25 (0.22 to 0.28)
Low middle	1,617.52 (1,427.99–1,826.03)	220.23 (193.29–247.91)	4,194.85 (3,622.48–4,785.99)	271.67 (235.41–309.31	0.60 (0.49 to 0.70)
Low	666.43 (563.64–771.96)	228.74 (192.186–266.75)	1,837.38 (1,599.79–2,101.74)	283.77 (248.2383–323.37)	0.67 (0.61 to 0.72)
World Bank Income level	11,673.11 (11,161.52–12,253.41)	275.27 (288.72–263.20)	20,609.55 (19,027.32–22,156.41)	247.58 (228.63–266.02)	−0.51 (−0.56 to −0.45)
World Bank High Income	4,604.68 (4,450.10–4,761.15)	383.00 (370.68–395.90)	4,998.86 (4,669.79–5,336.89)	259.14 (244.55–275.84)	−1.50 (−1.55 to −1.45)
World Bank Lower Middle Income	3,212.41 (2,934.61–3,522.53)	253.93 (232.08–279.24)	7,951.67 (6,978.48–8,885.18)	290.00 (255.10–323.87)	0.31 (0.22 to 0.39)
World bank upper middle income	3,405.14 (3,146.73–3,696.76)	200.48 (185.74–217.02)	6,556.36 (5,880.50–7,289.31)	190.77 (171.33–212.11)	−0.30 (−0.39 to −0.21)
World bank low income	450.88 (385.34–529.88)	250.92 (216.00–293.06)	1,102.66 (917.42–1,330.13)	285.27 (240.23–341.13)	0.41 (0.33 to 0.49)

DALYs, disability-adjusted life years; ASR-DALYs, age-standardized rate of disability-adjusted life years; EAPC, estimated annual percentage change; UI, uncertainty interval; CI, confidence interval.

**Table 3:** The cases number of years of life lost years lived with disability and their age-standardized rates of breast cancer in 1990 and 2019, and its temporal trends from 1990 to 2019.

**Characteristics**	**1990**				**2019**				**1990–2019**	
	**YLL** **No**. × **10**^3^ **(95% UI)**	**ASR–YLL** **per 100,000** **(95% UI)**	**YLD** **No**. × **10**^3^ **(95% UI)**	**ASR–YLD** **per 100,000** **(95% UI)**	**YLL** **No**. × **10**^3^ **(95% UI)**	**ASR–YLL** **per 100,000** **(95% UI)**	**YLD** **No**. × **10**^3^ **(95% UI)**	**ASR–YLD** **per 100,000** **(95% UI)**	**EAPC** **of ASR–YLL** **(95% UI)**	**EAPC** **of ASR–YLD** **(95% UI)**
Global	11,067.66 (10,628.42–11,579.86)	260.26 (249.82–272.07)	613.40 (431.91–830.18)	15.06 (10.63–20.28)	19,240.05 (17,814.22–20,770.36)	230.91 (213.83–249.12)	1,385.27 (971.37–1,873.27)	16.72 (11.74–22.56)	−0.56 (−0.62 to −0.50)	0.3 (0.28 to 0.35)
**Sex**										
Male	147.80 (136.61–160.16)	7.81 (7.22–8.44)	6.58 (4.63–8.75)	0.37 (0.26–0.49)	296.60 (261.48–328.53)	7.48 (6.60–8.26)	18.53 (13.23–25.06)	0.48 (0.34–0.64)	0.09 (−0.06 to 0.23)	1.1 (0.99 to 1.27)
Female	10,919.86 (10,484.22–11,440.52)	496.80 (476.86–520.17)	606.82 (427.20–821.21)	28.07 (19.80–37.92)	18,943.45 (17,533.33–20,455.08)	442.14 (409.03–477.52)	1,366.74 (956.85–1,845.10)	31.69 (22.17–42.81)	−0.56 (−0.63 to −0.50)	0.3 (0.32 to 0.40)
**SDI**										
High	3,392.75 (3,293.84–3,447.99)	345.51 (335.91–351.15)	321.05 (226.07–433.57)	32.03 (22.51–43.38)	3,569.30 (3,370.81–3,711.62)	221.63 (211.80–230.03)	514.55 (357.98–693.88)	31.06 (21.39–41.97)	−1.69 (−1.74 to −1.63)	−0.2 (−0.29 to −0.11)
High middle	3,009.69 (2,898.29–3,133.70)	271.72 (261.56–282.82)	159.73 (112.87–215.24)	14.63 (10.36–19.65)	4,209.03 (3,887.63–4,580.01)	212.27 (196.22–230.84)	361.22 (248.88–490.41)	18.16 (12.51–24.6)	−1.10 (−1.22 to −0.98)	0.7 (0.68 to 0.83)
Middle	2,426.28 (2,252.95–2,631.89)	195.72 (181.93–212.19)	79.76 (55.70–106.84)	6.69 (4.70–8.93)	5,601.21 (5,039.74–6,230.67)	207.80 (187.11–230.73)	322.15 (225.48–441.35)	12.047 (8.45–16.45)	0.17 (0.13 to 0.20)	2.1 (2.10 to 2.19)
Low middle	1,578.92 (1,396.79–1,780.26)	214.71 (188.56–241.79)	38.60 (27.08–51.42)	5.52 (3.89–7.37)	4,055.58 (3,497.02–4,633.46)	262.48 (227.39–299.51)	139.27 (100.01–187.97)	9.18 (6.60–12.30)	0.56 (0.46 to 0.67)	1.7 (1.62 to 1.81)
Low	652.51 (550.78–754.61)	223.67 (187.98–261.25)	13.92 (9.79–18.84)	5.06 (3.57–6.80)	1,790.13 (1,555.49–2,051.60)	276.19 (240.76–315.95)	47.25 (33.43–63.96)	7.58 (5.43–10.12)	0.65 (0.60 to 0.71)	1.3 (1.32 to 1.45)
World Bank Income level	11,060.06 (1,0621.27–11,572.22)	260.21 (249.78–272.03)	613.05 (431.66–829.69)	15.06 (10.63–20.28)	19,225.11 (17,801.60–20,754.39)	230.86 (213.78–249.06)	1,384.44 (970.77–1,872.13)	16.72 (22.56–11.74)	−0.56 (−0.62 to −0.50)	0.31 (0.28 to 0.35)
World bank high income	4,220.88 (4,099.68–4,284.12)	351.6 (342.19–356.77)	383.80 (269.93–520.57)	31.33 (22.02–42.62)	4,387.03 (4,146.70–4,567.73)	228.15 (218.01–237.17)	611.84 (428.33–828.73)	30.99 (21.50–41.99)	−1.66 (−1.71 to −1.60)	−0.13 (−0.23 to −0.03)
World bank lower middle income	3,129.37 (2,860.47–3,431.47)	247.03 (225.66–271.38)	83.04 (58.57–111.08)	6.90 (4.90–9.21)	7,682.18 (6,726.70–8,614.87)	279.98 (245.95–313.22)	269.48 (190–362.20)	10.02 (7.07–13.37)	0.28 (0.19 to 0.37)	1.21 (1.13 to 1.29)
World bank upper middle income	3,268.96 (3,008.66–3,555.23)	192.26 (177.38–208.22)	136.18 (95.83–184.25)	8.22 (5.80–11.09)	6,082.16 (5,460.54–6,795.06)	176.98 (158.97–197.61)	474.20 (326.66–648.66)	13.787 (9.49–18.80)	−0.43 (−0.53 to −0.33)	1.91 (1.85 to 1.98)
World bank low income	440.85 (377.38–516.22)	245.05 (210.81–284.57)	10.03 (7.02–13.62)	5.87 (4.12–7.89)	1,073.74 (893.29–1,295.41)	277.46 (233.99–331.63)	28.92 (19.99–39.63)	7.81 (5.47–10.54)	0.39 (0.32 to 0.47)	0.97 (0.89 to 1.06)

**Figure 1:**
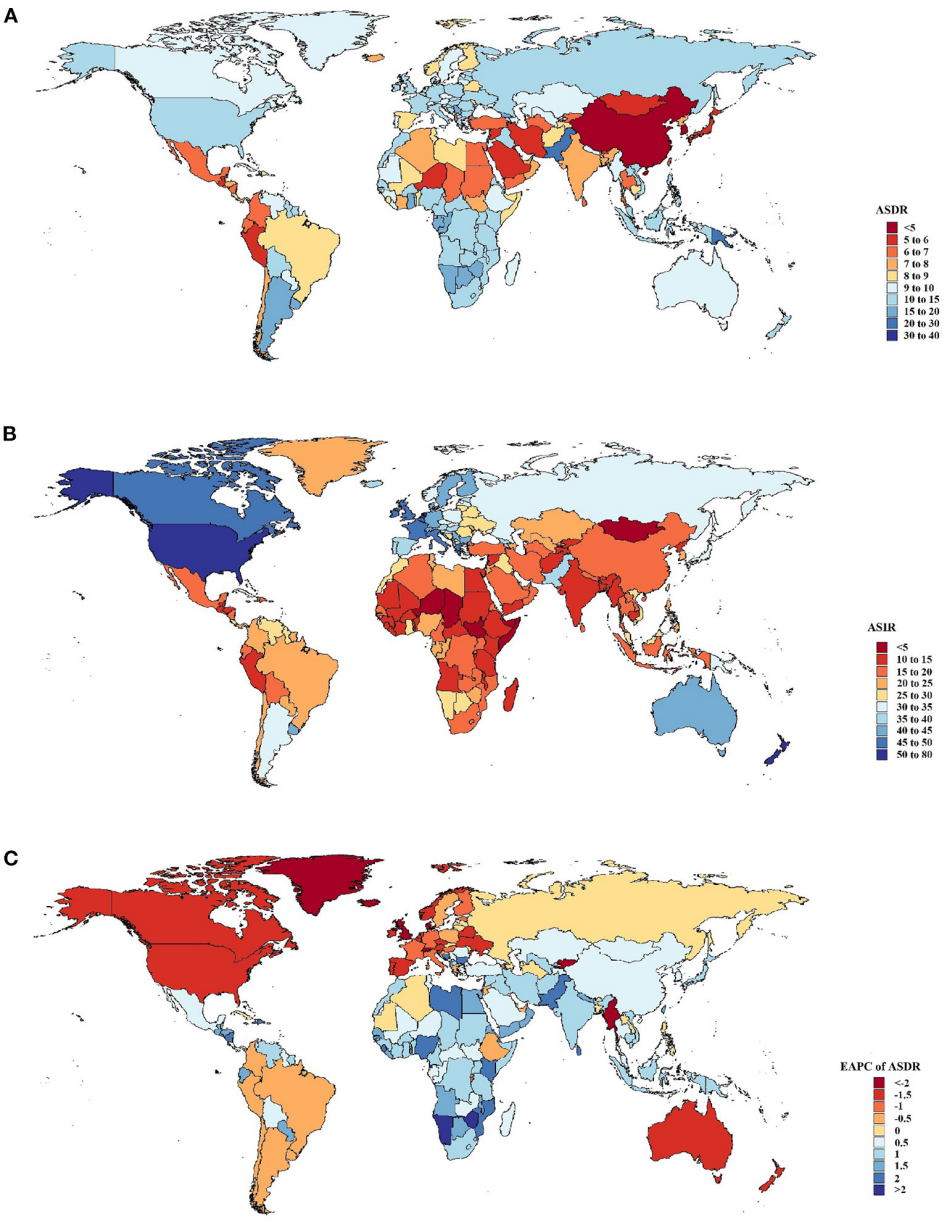
Global death and incidence cases and death rates of breast cancer from 1990 to 2019. **(A)** Age-standardized death rates (ASDRs) in 204 countries and territories in 2019. **(B)** Age-standardized incidence rates (ASIRs) in 204 countries and territories in 2019. **(C)** Estimated annual percentage changes (EAPCs) in ASDRs in 204 countries and territories from 1990 to 2019.

By country, deaths attributable to breast cancer increased most significantly in the Solomon Islands and the United Arab Emirates (Figure 1A). Compared to 1990, the percentage change increased separately by 1,169 and 851%. Furthermore, Qatar and Djibouti also observed a significant increase in breast cancer-related deaths (percentage change: 400–550%), whereas Denmark, Ukraine, Georgia, and Greenland observed decreases (percentage change: 15–30%). Moreover, the ASDR was observed in the Solomon Islands, where it was much higher than elsewhere (37.1 [95% UI: 29.18–46.81]). Moreover, the highest ASIRs were found in Monaco (78.51 [95% UI: 57.30–101.60]) (Figure 1B). In contrast, the lowest ASDRs were found in Korea and China in 2019 (4–5 per 100,000 population). The fastest ASDR declines were seen in countries with relatively high SDIs, such as Denmark and Greenland, with EAPCs ranging from as low as −3.64 to −2.12 (Figure 1C). Over the past few decades, the Solomon Islands exhibited a high ASDR and the sharpest increase in ASDR, which may indicate that healthcare is a significant challenge. Uzbekistan not only had the highest ASDR in the world but also exhibited the sharpest increase in ASDR.

### 4.2. Age-wise distribution of breast cancer in developing regions

**Figure 2:**
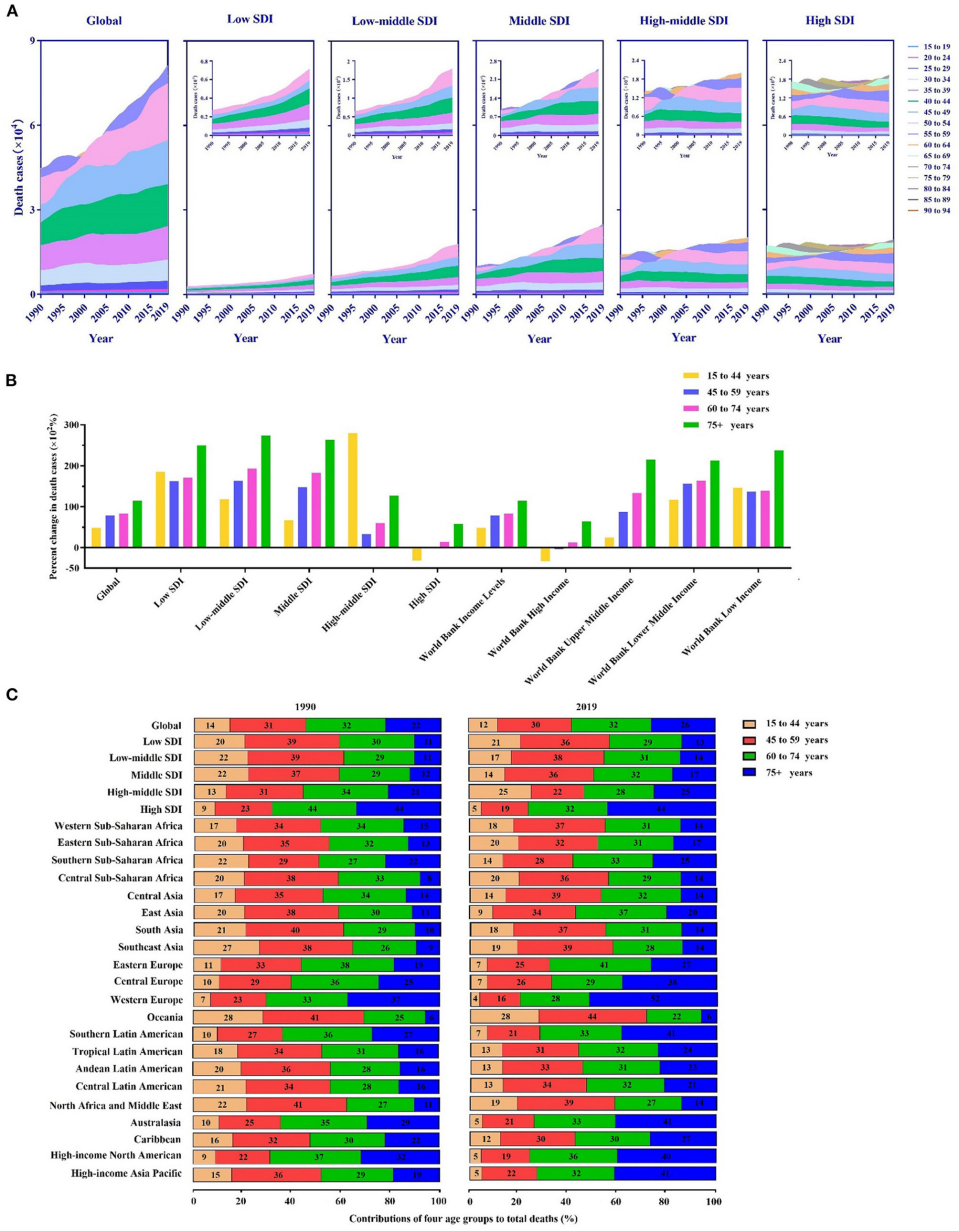
Death cases of breast cancer and their changes by age groups in different regions. **(A)** The contribution of each age group (15–94 years) to the total number of deaths between 1990 and 2019 globally and in territories with a low to high sociodemographic index (SDI). **(B)** Percentage changes in deaths in the four age groups (15–44 years, 45–59 years, 60–74 years, and 75–94 years) between 1990 and 2019 globally and in territories with a low to high SDI. **(C)** The four age groups as percentages of the total number of deaths globally and in territories with a low to high SDI and in 21 global burdens of disease (GBD) regions in 1990 and 2019.

As shown in Figure 2A, an increasing trend in the global breast cancer burden was observed with age. Remarkably, most breast cancer deaths occurred in the 35–54-year age group. In addition, all age groups showed a marked increase in deaths in the low-to-mid SDI regions, whereas they remained stable between 1990 and 2019 in the high SDI and high-middle SDI regions.

The population was divided into four age groups: 15–44 (youth), 45–59 (middle-aged), 60–74 (middle-old), and 75–94 years (elderly). Our analysis and comparison of a percentage change in deaths by the group in different SDI regions from 1990 to 2019 were performed (Figure 2B). Overall, the elderly had the highest increase in deaths, followed by middle-aged people. The change in deaths in the 15–44-year age group was highest in the high-middle SDI region. Values in the high SDI region were in the negative range. As a result, compared with the population in developed areas, mortality rates in developing regions increased faster and significantly in the middle-aged and elderly groups. Meanwhile, the long-term burden of breast cancer was increasing. We also observed the same trend at the WB income level, and the younger group showed negative values in high-income regions. Low-income regions had the most significant change in mortality among the elderly.

From 1990 to 2019, the proportion of breast cancer deaths among the elderly was higher in the high SDI region than in the low SDI region (Figure 2C). Among all GBD regions, Oceania had the youngest age distribution; young and middle-aged groups accounted for 69% of deaths in 1990 and 72% in 2019. Conversely, Western Europe had the oldest distribution, accounting for 37% of deaths in 1990 and 52% of deaths in 2019. Additionally, only Western Sub-Saharan Africa experienced a decline in the age of death.

### 4.3. Risk factors contribute to the majority of breast cancer-related deaths in developing countries

**Figure 3:**
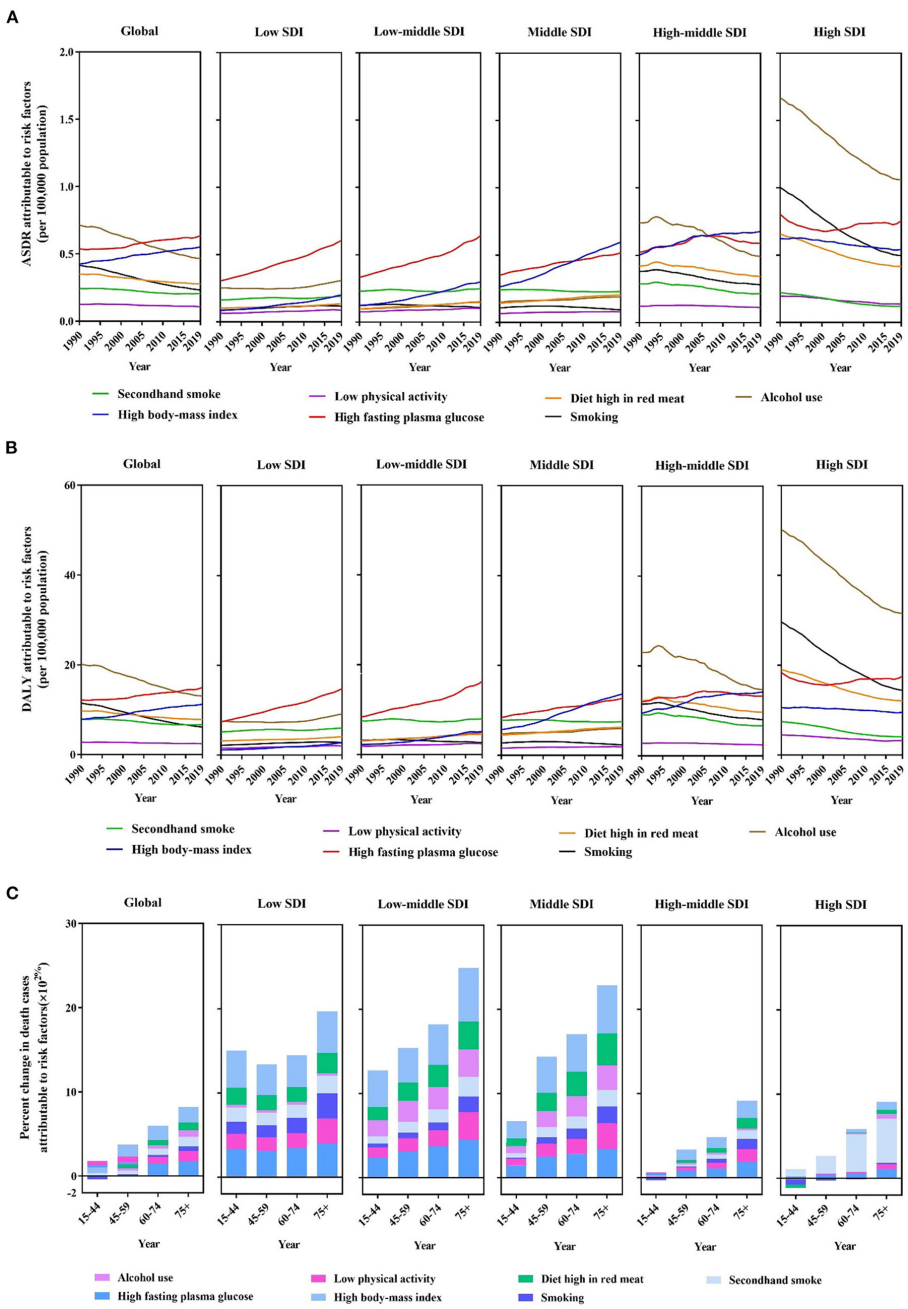
A predominant contribution of risk factors to breast cancer-related deaths by SDI regions and age groups. **(A)** ASDRs attributable to main risk factors by SDI regions from 1990 to 2019. **(B)** Disability-adjusted life years (DALYs) attributable to main risk factors by SDI regions from 1990 to 2019. **(C)** Percentage changes in deaths attributable to four metabolic risk factors by age groups and SDI regions between 1990 and 2019.

The burden of breast cancer is primarily due to metabolic and behavioral risk factors in the global disease burden database. ASDRs and DALY are attributable to seven risk factors, namely alcohol consumption, high BMI, high FPG, a diet high in red meat, low physical activity, smoking, and secondhand smoke. Compared with the risk factors for ASDR in 1990, smoking and alcohol consumption decreased. In contrast, high FPG and high BMI were ranked as significant contributors to increased ASDR in 2019 (Figure 3A). These two risk factors have increased rapidly in low SDI, low-middle SDI, and middle SDI regions over the past three decades. Significantly, high BMI became the top ASDR risk factor in 2019 in middle and high-middle SDI regions. Although alcohol consumption, which contributes to ASDRs, decreased in high SDI regions, it was the leading cause of death in 2019. Furthermore, other factors are relatively stable, and changes are not clear. Deaths attributed to DALYs followed the same pattern as risk factors (Figure 3B). Death rates attributable to high FPG increased considerably in low and low-middle SDI regions but tended to remain flat in high-middle and high SDI regions. Furthermore, between 1990 and 2019, the number of DALYs due to alcohol consumption in breast cancer decreased globally, and this trend is consistent in high and high-middle SDI regions.

In the global level, there was a tremendous increase in breast cancer mortality rate among the populations with high FPG and high BMI in the middle-aged to elderly groups (Figure 3A). The percentage changes in deaths due to the seven risk factors in low to middle SDI regions increased significantly over time across all age groups. Moreover, a diet high in red meat and a high BMI are the largest increases in magnitude. Meanwhile, in the 15–44-year age group, smoking exhibited nearly negative trends in the high-middle and high SDI regions. Similarly, alcohol consumption and high BMI exhibited negative trends in the high SDI region in the youngest group (Figure 3C).

**Figure 4:**
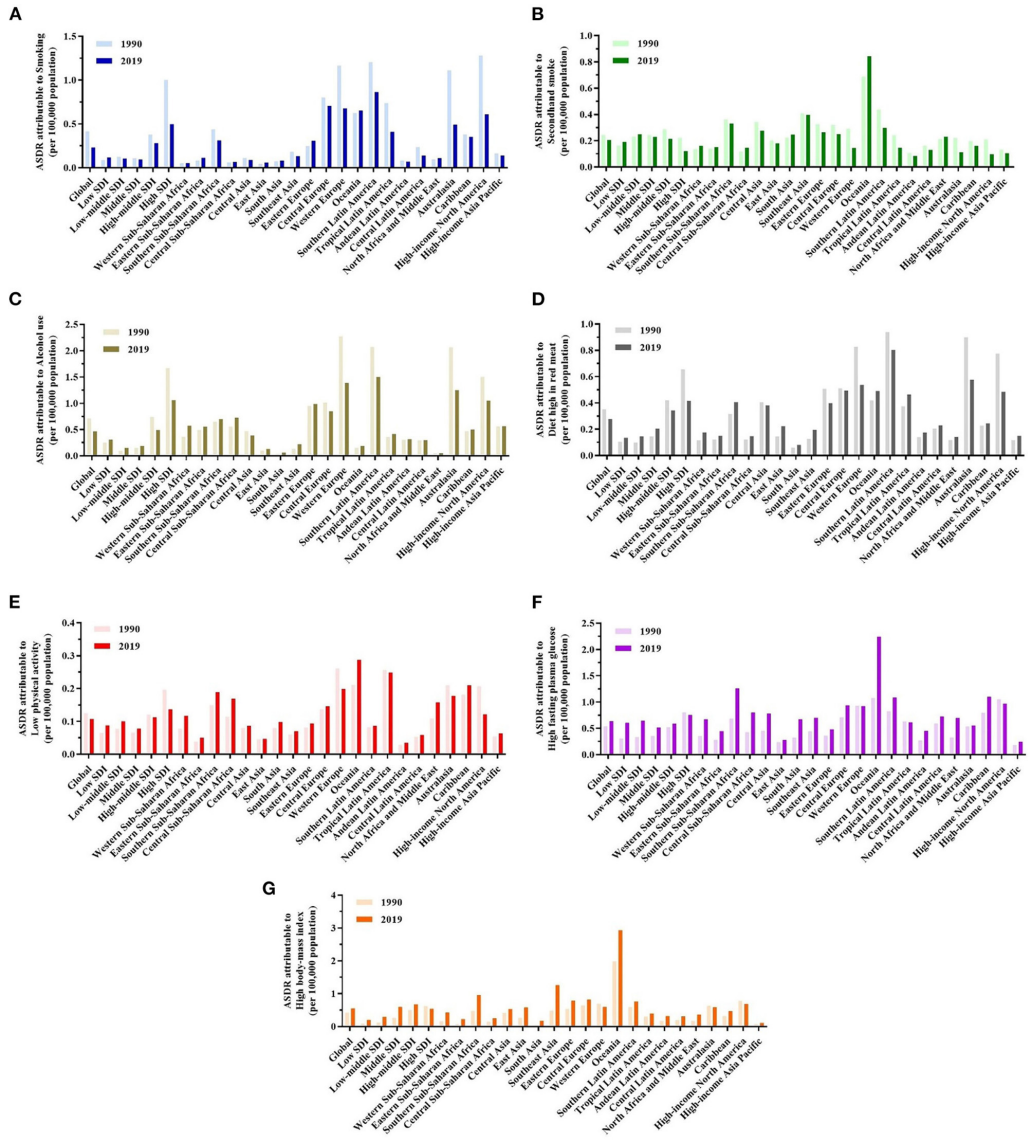
A predominant contribution of risk factors to breast cancer-related deaths in different regions and territories with a low to high SDI and in 21 GBD regions in 1990 and 2019. **(A–G)** ASDRs attributable to smoking, secondhand smoke, alcohol consumption, a diet high in red meat, low physical activity, high fasting plasma glucose (FPG), and high body mass index (BMI).

In 2019, ASDRs attributable to behavioral risk factors were declining globally (Figures 4A–E). However, at the geographical level, smoking increased in Eastern Sub-Saharan Africa, Eastern Europe, Oceania, North Africa, and the Middle East regions. On the other hand, developed regions, such as Western Europe, Southern and Tropical Latin America, Australasia, and high-income North America experienced significant reductions in ASDR attributable to secondhand smoke; nevertheless, Oceania experienced an increase during this time. ASDR attributable to a diet high in red meat increased in most developing regions, such as Africa, Latin America, and Asia. ASDR trends attributable to alcohol consumption have increased similar to a diet high in red meat; however, it is worth noting that South Latin America has declined in 2019.

Globally and in major GBD regions, metabolic risk factors contributed to increased deaths (Figures 4F, G). Notably, Oceania had the highest ASDRs attributable to high FPG and high BMI in 2019. The GBD regions in Southeast Asia and Southern Sub-Saharan Africa had the highest burden of high BMI. Southern Sub-Saharan Africa also increased rapidly, resulting in high FPG.

## 5. Discussion

According to the 2019 GBD Study dataset, which compares mortality, sex, and age groups across regions, countries, ages, and timelines, this study offers an updated analysis of the global burden and risk factors for breast cancer. Globally, the number of breast cancer incidents increased from 0.88 million in 1990 to 2 million in 2019. Furthermore, ASIR changes increased by 12%. Globally, deaths from breast cancer increased from 0.38 million in 1990 to 0.7 million in 2019. However, global ASDR has been declining continuously since 1990, suggesting that aging and population growth may have contributed to this increase.

Our results showed that EAPC was positively associated with ASIR and ASDR in low to middle SDI regions. It indicates that some areas showed rapid growth. From the WB income level, EAPC was positively associated with ASIR and ASDR in the WB's low- and lower- and middle-income levels in the period between 1990 and 2019. The results of this study suggest that breast cancer prevention and treatment programs should not be ranked below other public health problems in those countries. For example, The Solomon Islands experienced high ASDRs for decades and a sharp increase, indicating a significant healthcare concern. The situation was especially perilous in Uzbekistan, where ASDR increased rapidly and was the highest in the world.

In terms of the overall age distribution, most breast cancer deaths were among people between the ages of 35–54. Compared with developed areas, deaths in developing regions increased faster and significantly in the middle-aged to elderly population. Meanwhile, the long-term burden for breast cancer had been growing. A similar trend was observed for the WB income level. The younger group showed negative values in high-income regions. Low-income regions experience the most significant change in deaths among the elderly, while breast cancer awareness among the general public and healthcare workers was poor in low-income countries (17). However, according to a recent microsimulation study, raising awareness and improving breast cancer treatment in low- and middle-income countries can reduce mortality rates and improve survival rates (18). Mammographic screening is also a major contributor (19). In some Asian countries, women below 50 years are at a particularly high risk of breast cancer. Due to an increasing number of behavioral risk factors, such as late marriage, low equality rates, and short breastfeeding periods, the mortality rate from the disease is increasing in transition countries (20–22). Among of all GBD regions, Oceania had the youngest proportion of breast cancer-related deaths. Additionally, there was a downward trend of the age at death only in Western Sub-Saharan Africa. Nevertheless, there is no change in the age of the middle and middle-old population. Breast cancer global burden is affected by a variety of adjustable factors including parity, alcohol consumption, BMI, and contraceptive use, as well as non-adjustable factors, including family history, age, and menopause (23–25). This study presents an analysis of seven major risk factors for breast cancer death and DALYs, including alcohol consumption, high FGB, high BMI, a diet high in red meat, low physical activity, secondhand smoke, and smoking.

Meanwhile, high BMI and high FGB were both found to be potential risk factors for breast cancer deaths and DALYs. Globally, these two risk factors showed an increasing contribution trend, with rapid increases from 1990 to 2019 in low, low-middle, and middle SDI regions. Irregular menstruation or fewer ovulatory cycles may contribute to breast cancer risk and may be inversely associated with BMI (26). In addition, premenopausal women with a high BMI have lower estrogen levels because estradiol is substantially absorbed into fat, and estrogen is cleared from the liver more rapidly (27). As a result, controlling weight improves overall health (28). In addition to the abovementioned risks, dense breast tissues in obese women increases the risk of breast cancer in postmenopausal women (29). According to the study (30), high-income Western countries, Central Asia, the Middle East, and North Africa suffered the most significant increase in obesity. The reason for the increase may be due to changes in body systems around the globe that promote high-calorie, low-nutrient foods and reduced physical activity. As the BMI increases, breast cancer risk increases, and Asians have a higher risk than North Americans and Europeans. According to the research, older women and those born more recently had high incidence rates in China. There may be a connection between these trends and lifestyle patterns, such as changes in diet, decreased physical activity, and increased obesity (9). For example, the prevalence of obesity in Chinese adults tripled between 2004 and 2014, according to National Chronic Disease and Risk Factor Surveillance data (31).

It has been demonstrated that alcohol consumption may contribute to the risk of breast cancer (32–34). In addition, breast cancer may be associated with alcohol consumption on a dose–response basis (35). Furthermore, alcohol consumption has been linked to increased breast density (36). Although daily alcohol consumption has declined significantly worldwide in the past three decades, alcohol consumption is increasing in most developing countries, such as Africa and Asia. In addition, we used the GBD data to examine the influence of diets with high red meat on breast cancer mortality. It has been observed that the ASDR trend associated with a diet high in red meat is similar to alcohol consumption. However, a low-fat diet significantly reduces the risk of death from breast cancer (37). Therefore, the westernization of lifestyle contributed to an increased risk of breast cancer-related deaths among women in lower-SDI quintiles through a diet high in red meat. In another study, healthy lifestyles were shown to lower the risk of breast cancer (38).

Current and former smokers both have a higher risk of breast cancer than non-smokers, according to a meta-analysis study (39). Furthermore, in 2019, developing regions such as Eastern Sub-Saharan Africa, Eastern Europe, Oceania, North Africa, and the Middle East regions experienced an increase in ASDRs attributable to smoking. Moreover, during the same period, Oceania experienced a significant increase in ASDRs attributable to secondhand smoke.

In recent years, higher SDI regions experience a reduction in disease burden, while lower SDI regions experience an increase in breast cancer burden. Over time, disparities could widen. Furthermore, it is particularly important to prevent the acceleration of these disparities. In addition, income levels, education, and health insurance status need to be improved, which may affect healthcare access and breast cancer stage at diagnosis. Changes in reproductive patterns, such as having the first baby at a younger age, having increased parity, using less hormone replacement therapy (HRT) during menopause, and extending breastfeeding for a longer period, are also measures that need to be taken. People should also reduce smoking and drinking, exercise regularly, eat healthy food, stay in shape, and have a healthy lifestyle (40). Non-genetic, modifiable risk factors should be emphasized in countries with lower SDIs, and steps should be taken to address attributable risk factors to reduce the prevalence of breast cancer.

This study examined and revealed the latest trends and patterns in global breast cancer incidence, mortality, DALYs, and the most relevant risk factors, particularly in developing countries, over the period of 1990–2019. Unlike other previous studies, this study updated the global burden data of breast cancer. The results analyzed the changes in trends in disease burden not only from a global scale but also from different social development index regions, different WB income levels, and 21 GBD countries over the past three decades. Furthermore, four different age groups were analyzed in detail. Seven risk factors attributable to breast cancer disease burden were also analyzed in depth from the perspectives of age stratification and geographic regions. This study summarizes breast cancer trends in developing countries and provides more targeted guidance for formulating public health policies.

## 6. Conclusion

In low- and medium-income regions (41), breast cancer awareness and breast cancer misperceptions must be addressed in public health policies. The development and adoption of cost-effective screening and therapeutic solutions, mitigation of risk factors, and establishment of a cancer infrastructure are essential. In addition, countries should assess their breast cancer burden and establish strategic priorities to reduce mortality and morbidity based on specific data on local prevalence. Regular updates on disease conditions within the GBD framework can be very helpful in providing timely assessment and guidance to address these serious disease problems.

### 6.1. Limitations

However, the 2019 GBD Study's detailed and high-quality analysis of GBD and risk factors for death has several limitations. Data collected by various regions and countries may vary in quality, comparability, accuracy, and degree of omission. Some deviations inevitably occur in the estimated values, even with many statistical methods. Furthermore, variations in mortality and incidence may be partly explained by changes in screening protocols rather than changes in age-specific rates.

## Data availability statement

The original contributions presented in the study are included in the article/supplementary material, further inquiries can be directed to the corresponding author.

## Author contributions

LL and BZ: data curation (Equal). JK: writing-review and editing (Supporting). SL: supervision (Supporting). HW: conceptualization, supervision, and writing-review and editing (Lead). All authors contributed to the article and approved the submitted version.
